# Measuring instrumental activities of daily living in non-demented elderly: a comparison of the new performance-based Harvard Automated Phone Task with other functional assessments

**DOI:** 10.1186/s13195-018-0464-x

**Published:** 2019-01-10

**Authors:** Gad A. Marshall, Sarah L. Aghjayan, Maria Dekhtyar, Joseph J. Locascio, Kamal Jethwani, Rebecca E. Amariglio, Sara J. Czaja, David A. Loewenstein, Keith A. Johnson, Reisa A. Sperling, Dorene M. Rentz

**Affiliations:** 1 Center for Alzheimer Research and Treatment, Department of Neurology, Brigham and Women’s Hospital, Harvard Medical School, Boston, MA 02115 USA; 2 Massachusetts Alzheimer’s Disease Research Center, Department of Neurology, Massachusetts General Hospital, Harvard Medical School, Boston, MA 02114 USA; 3 Connected Health Innovation, Partners HealthCare, Harvard Medical School, Boston, MA 02114 USA; 4 Department of Psychiatry and Behavioral Sciences, Center on Aging, University of Miami Miller School of Medicine, Miami, FL 33136 USA

**Keywords:** Alzheimer’s disease, Instrumental activities of daily living, Everyday functioning, Mild cognitive impairment, Performance-based, Validation

## Abstract

**Background:**

Impairment in instrumental activities of daily living (IADL) may occur in the earliest stages of mild cognitive impairment (MCI). However, there are few reliable measures of IADL in MCI or that have a sufficient range of scores in clinically normal (CN) elderly. The objective of this pilot study was to examine the convergent validity of a phone performance-based IADL instrument, the Harvard Automated Phone Task (APT), designed to measure the earliest IADL changes in Alzheimer’s disease (AD), with other sensitive performance-based and subjective measures of everyday functional capacity among CN and MCI participants.

**Methods:**

Twenty-nine CN and 17 MCI participants were administered the Harvard APT, the computer performance-based Czaja Functional Assessment Battery (CFAB), and the AD Cooperative Study ADL prevention instrument (ADCS ADL-PI) participant and study partner versions; in addition, 52 different CN and 7 MCI participants were administered the Harvard APT and the Subjective Study Partner and Participant-reported (SSPP) IADL scale. The Harvard APT was compared with the three other IADL assessments.

**Results:**

In both CN and MCI, better performance on the Harvard APT was associated with better performance on the CFAB. In CN, better performance on the Harvard APT was associated with better ADCS ADL-PI participant-reported IADL, while in MCI better performance on the Harvard APT was associated with better ADCS ADL-PI study partner-reported IADL. Furthermore, in CN better performance on the Harvard APT was associated with better SSPP-IADL participant and study partner-reported IADL.

**Conclusions:**

In this small pilot study, the Harvard APT, a brief, self-administered, objective measure of IADL performance, appears to correlate well with other sensitive measures of everyday functioning, providing good preliminary convergent validity for this new measure. Moreover, it appears to perform well across both CN and MCI participants, which suggests that it is a promising measure of early, clinically meaningful functional change. This may not be the case as suggested in our small sample for subjective IADL scales that may perform differentially depending on the reporter (self vs. study partner) across the clinical spectrum possibly due to diminishing awareness of IADL difficulties in individuals who become cognitively impaired. Secondary prevention trials in AD have a great need for such ecologically valid and reliable measures of early IADL changes.

**Electronic supplementary material:**

The online version of this article (10.1186/s13195-018-0464-x) contains supplementary material, which is available to authorized users.

## Background

Instrumental activities of daily living (IADL) consist of complex everyday activities such as managing medications and finances, driving, and doing household chores. Impairment in the performance of IADL can occur with normal cognitive aging and can also begin at the stage of mild cognitive impairment (MCI) [1–3]. However, there are few reliable measures that can detect changes in IADL performance in individuals with MCI and, more importantly, detect such performance changes in clinically normal (CN) elderly individuals who may be at risk for Alzheimer’s disease (AD) [4].

In anticipation of upcoming secondary prevention trials in preclinical AD and ongoing treatment trials in prodromal AD (MCI), the Food and Drug Administration (FDA) issued new guidelines for outcome measures in early-stage AD clinical trials [5, 6]. For MCI trials, the FDA recommended a combined cognitive and IADL measure such as the Clinical Dementia Rating (CDR) scale or two separate measures, one for cognition and the other for IADL, whereas for prevention trials they recommended a single sensitive cognitive outcome measure. However, they added that following the initial success of a prevention trial with a cognitive outcome, a more clinically relevant outcome such as long-term evidence of IADL benefit would be required. Since currently there are few well-validated sensitive IADL measures for preclinical AD and no clear gold standard, the FDA did not recommend including a primary IADL outcome measure in prevention trials.

Recently, in an attempt to fill this gap, our group has developed a new phone performance-based IADL measure targeting CN elderly at risk for AD as they transition to MCI. This novel test called the Harvard Automated Phone Task (APT) consists of navigating an interactive voice response system (IVRS) to complete daily tasks such as refilling a prescription and contacting one’s health insurance company [7, 8]. The telephone is still the most commonly used technological modality of communication among the elderly in North America, many of whom regularly engage in tasks requiring an IVRS; these tasks are challenging for the elderly and therefore can potentially help detect early IADL changes [9]. The tasks selected, while not encompassing many IADL, were meant to reflect medical- and financial-related activities, which are both often challenging to the elderly and have innate clinical relevance.

Many IADL scales currently in use have limited information on their psychometric properties [10, 11] or rely on participant or study partner judgments, which may introduce potential reporter bias when IADL impairment is noted [12]. Therefore, it is imperative to further validate and characterize existing and new IADL tests, particularly those which provide objective measures of functional performance. In a larger sample (*n* = 207), we previously found that the Harvard APT discriminated well between diagnostic groups (young normal, CN elderly, and MCI) in relation to information processing speed and executive function at baseline, and had adequate test-retest reliability/stability; these results were independent of age, education, hearing acuity, and motor speed [7]. The Harvard APT also tracked well with global cognition, information processing speed, executive function, and memory over time [8].

The objective of the current pilot study was to assess the convergent validity of the Harvard APT with other IADL measures, specifically comparing it with performance-based and subjective (participant and study partner-reported) IADL tests in both CN and MCI participants. We compared the Harvard APT to another recently developed and validated computer-based functional performance battery, the Czaja Functional Assessment Battery (CFAB) [13, 14], as well as with an established subjective IADL scale, the Alzheimer’s Disease Cooperative Study ADL prevention instrument (ADCS ADL-PI) [15], and another recently developed and yet to be validated subjective IADL scale, the Subjective Study Partner and Participant-reported IADL (SSPP-IADL) scale. We explored performance on these measures within a cohort of CN and MCI participants who may be at risk for AD. The nature of our analyses was exploratory to compare multiple aspects of the Harvard APT with the other IADL tests. Convergent validity is an important psychometric property for a test which is often not assessed or reported, especially for IADL assessments targeting individuals at risk for early-stage AD. Examining these different instruments provides an opportunity to compare performance-based versus subjective measures and compare the modality of administration—the computer or tablet vs. the phone—which is more commonly used by the elderly. Moreover, the subjective IADL assessments used in this study include participant (self) and study partner (informant) reports, which will give us the opportunity to look for different association patterns based on the reporting source in this sample of CN and MCI participants. We hypothesized that the primary outcome reported for the Harvard APT will have moderate to strong correlations with the primary outcomes reported for the other performance-based and subjective IADL tests. Moreover, the Harvard APT may have the advantage of being a sensitive measure across both CN and MCI participants when administered directly to the participant, whereas the subjective IADL scales may have differential sensitivity depending on administration to participant vs. study partner in CN vs. MCI. We are not aware of a precedent for describing this differential sensitivity with IADL tests. However, a similar phenomenon has been seen with subjective cognitive concerns when comparing participant and study partner report of cognitive concerns to objective cognitive assessment. This is presumably due to a growing lack of awareness in individuals with MCI [16].

## Methods

### Participants

For the comparison of the Harvard APT with the CFAB and ADCS ADL-PI, 29 CN elderly participants were recruited from the community, and 17 amnestic MCI participants were recruited from the Brigham and Women’s Hospital (BWH) and Massachusetts General Hospital (MGH) memory disorders clinics, and the Massachusetts Alzheimer’s Disease Research Center (MADRC). For the comparison of the Harvard APT with the SSPP-IADL, 52 CN and 7 MCI participants were recruited separately from the same sources at an earlier time point; these participants had comparable demographics. All participants were in generally good physical health or had stable chronic medical conditions. None of the participants had active major psychiatric disorders (such as major depressive disorder, bipolar disorder, or schizophrenia).

CN participants were aged 60 to 87 years old (inclusive), had a Mini-Mental State Examination (MMSE) [17] score of 25 to 30 (inclusive), and normal memory performance (defined as a Free and Cued Selective Reminding Test (FCSRT) [18] free recall score of > 24 and cued recall score of > 44). MCI participants were aged 63 to 89 (inclusive), had an MMSE score of 24 to 29 (inclusive), impaired memory performance (all participants had a FCSRT free recall score of ≤ 24; 8 participants had a FCSRT cued recall score of 48, and 9 participants had a cued recall score of < 44), and did not meet the National Institute on Aging—Alzheimer’s Association criteria for dementia [19].

The Partners Healthcare Institutional Review Board approved the study. Written informed consent was obtained from all participants prior to initiation of any study procedures in accordance with Institutional Review Board guidelines.

### Clinical assessments

#### The Harvard Automated Phone Task (APT)

As previously described [7, 8], the Harvard APT consists of navigating an IVRS on the phone to complete the following three tasks: 1) APT-Script, refilling a prescription; 2) APT-PCP, calling a health insurance company to select a new primary care physician; and 3) APT-Bank, making a bank account transfer and payment. Due to inadequate data, APT-Bank was not included in the current analyses.

Tasks are scored based on total time (until disconnected), number of errors, number of repetition of steps (when a participant prompts the system to repeat the last recording), and correct completion of task (dichotomous variable). The measure of interest used for each of the tasks in the current analyses is total time, adjusted for correct completion (as previously described [7, 8]) for participants who did not complete the task correctly (time was adjusted to reflect noncompletion with greater resulting time values by assigning a total time equivalent to the longest total time among individuals who correctly completed the task). Higher values of adjusted time indicate worse performance.

#### The Czaja Functional Assessment Battery (CFAB)

The CFAB is a newly developed computer-based measure of everyday functional capacity that includes simulations of everyday activities [13, 14]. In a larger sample, the CFAB discriminated well between CN elderly and MCI in relation to cognitive function, and had good test-retest reliability [14]. The CFAB is currently available in English and Spanish. In the version of the CFAB used in the current study, individuals used a touch screen tablet or computer to complete the following two tasks: 1) CFAB-Script, refilling a prescription using a simulated IVRS; and 2) CFAB-ATM, performing automated teller machine (ATM) transactions. Tasks are scored based on total time to completion, number of correct items, number of errors, and rate (number of correct items/total time). The measures used in this study for each of the two tasks were total time (higher values of time indicate worse performance) and rate, which is a measure of task efficiency (lower values of rate indicate worse performance). Participants completed the CFAB on the same day as the Harvard APT.

#### The Alzheimer’s Disease Cooperative Study ADL prevention instrument (ADCS ADL-PI)

The ADCS ADL-PI is a subjective IADL scale used in AD prevention trials [15]. In a larger sample, it has been shown to discriminate well between CN elderly and MCI and predict future cognitive decline in CN [15]. It consists of 18 items addressing performance of IADL over the past 3 months that are administered separately to the participant and a study partner such as a family member or close friend. Each item is scored on a scale of 1 to 3: a score of 1 indicates they perform the activity as well as usual, with no difficulty; 2 with a little difficulty; and 3 with a lot of difficulty. Participants or study partners can also indicate that the participant did not perform the given activity or that the study partner does not know if the participant performed the given activity; for such instances, the given item is not scored. An average score of all items was used in the current analyses with a higher score indicating greater impairment. Participants and study partners completed the ADCS ADL-PI on the same day as the Harvard APT.

#### Subjective Study Partner and Participant-reported instrumental activities of daily living (SSPP-IADL) scale

We developed a new subjective IADL scale, the SSPP-IADL. We employed two large databases (the Alzheimer’s Disease Neuroimaging Initiative and the MADRC longitudinal cohort) to assess which items of three existing scales (the Functional Assessment Questionnaire (FAQ) [20], the Structured Interview and Scoring Tool—MADRC (SIST-M) [21], and the Everyday Cognition (ECog) [22]) best discriminate between CN elderly and MCI and predict disease progression from CN to MCI. In separate analyses, we assessed the 10 FAQ items, 41 IADL-related SIST-M items, and 17 IADL-related ECog items using multivariate linear discriminant, logistic regression, and Cox proportional hazards regression analyses [23–25]. We found 12 items that either significantly distinguished between CN and MCI (10 items) or predicted progression from CN to MCI (four items, two of which overlapped with the previous 10 items). We also developed six new items that primarily target technology-dependent activities since these are becoming part of daily life and have not been assessed adequately in prior subjective IADL scales (see Additional file 1: Table S1).

The complete SSPP-IADL scale consists of 18 items addressing performance of IADL over the past 3 years that are administered separately to the participant and a study partner. Each item is scored on a scale of 0 to 5: a score of 0 indicates no change; 1 takes longer to do than in the past; 2 has difficulty, but can do by oneself; 3 requires prompting; 4 requires assistance; and 5 dependent/cannot do. Participants or study partners can also indicate that the participant never performed the given activity; for such instances, the given item is not scored. An average score of all items was used in the current analyses with a higher score indicating greater impairment. Participants and study partners completed the SSPP-IADL on the same day as the Harvard APT.

### Statistical analyses

Analyses were performed using SAS version 9.4 (SAS, Cary, NC) and SPSS version 24.0 (IBM). CN and MCI participant demographics and characteristics were compared using *t* tests and Chi-square tests and *p* values were reported (see Table 1).

**Table 1:** Demographics and characteristics of participants

	CN	MCI	*p* value
*n*	29	17	
Age (years)	75.6 ± 7.6 (60–87)	78.6 ± 7.9 (63–89)	0.20
Sex (% female)	72.4	47.1	0.09
Education (years)	16.2 ± 3.1 (6–20)	16.6 ± 2.1 (12–20)	0.66
AMNART IQ	121.2 ± 11.3 (80–131)	121.6 ± 9.1 (99–131)	0.90
MMSE	28.9 ± 1.5 (25–30)	27.6 ± 1.7 (24–29)	0.007
FCSRT free recall	34.2 ± 4.3 (26–41)	13.8 ± 8.0 (3–24)	< 0.001
FCSRT cued recall	47.8 ± 0.5 (46–48)	37.1 ± 12.8 (13–48)	0.003

Demographics and characteristics for CN and MCI participants were compared using *t* tests for continuous variables and Chi-square tests for categorical variables, and *p* values are reported

*AMNART IQ* American National Adult Reading Test intelligence quotient, *CN* clinically normal, *FCSRT* Free and Cued Selective Reminding Test, *MCI* mild cognitive impairment, *MMSE* Mini-Mental State Examination

Spearman correlations were used to test the associations between the Harvard APT (APT-Script and APT-PCP adjusted time), CFAB (CFAB-Script time, CFAB-Script rate, CFAB-ATM time, CFAB-ATM rate), and participant-reported and study partner-reported ADCS ADL-PI and SSPP-IADL average scores in CN and MCI participants separately, as well as across all participants. Correlation coefficients and *p* values were initially reported without adjustment for multiple comparisons. We then reported results of false discovery rate (FDR) correction for multiple comparisons. Since the distribution of the Harvard APT is not normal, Spearman’s rank-order (nonparametric) correlations were performed. These results are presented in Table 3.

General linear models were used to further test the associations between APT-Script and APT-PCP adjusted time (dependent variables in separate models) and CFAB or ADCS ADL-PI variables and their interaction with diagnosis (predictors in separate models: CFAB-Script time, CFAB-Script rate, CFAB-ATM time, CFAB-ATM rate, participant-reported and study partner-reported ADCS ADL-PI). SSPP-IADL data were not analyzed because too few MCI participants were administered this scale. Partial unstandardized regression coefficient estimates (β) with 95% confidence intervals (CI) and significance test results (*p* values) were reported.

A factor analysis was performed on the IADL measures across CN and MCI participants to determine the dimensionality of these measures, that is whether their relations were consistent with a single underlying construct or not. We included the following variables in this analysis: APT-Script and APT-PCP adjusted time, CFAB-Script and CFAB-ATM time, participant-reported and study partner-reported ADCS ADL-PI. CFAB rate variables were not included to avoid mathematical artifacts owing to their being computed partly as a function of the time variables (rate = number of correct items/total time). The SSPP-IADL was not included due to a lack of overlap with participants who underwent the CFAB. We ran an oblique (correlated) factor analysis with a promax rotation, based on a pairwise deleted correlation matrix (to provide maximal information).

## Results

Participant demographics and characteristics for those undergoing the Harvard APT, CFAB, and ADCS ADL-PI are provided in Table 1. As expected, MCI participants performed worse than CN participants on measures of cognition (MMSE and FCSRT). Participants who underwent the Harvard APT and SSPP-IADL had comparable demographics and characteristics. Table 2 shows performance by CN and MCI participants for the different IADL tests (Harvard APT, CFAB, ADCS ADL-PI, and SSPP-IADL). Spearman correlations between the Harvard APT and other IADL tests are presented in Table 3.

**Table 2:** Performance by CN and MCI participants on the Harvard APT, CFAB, ADCS ADL-PI, and SSPP-IADL

			CN	MCI
Harvard APT	APT-Script	Adjusted time	66.4 ± 20.9	75.3 ± 38.4
	APT-PCP	Adjusted time	226.0 ± 117.8	346.2 ± 141.3
CFAB	CFAB-Script	Time	222.8 ± 88.8	246.7 ± 107.0
		Rate	0.054 ± 0.019	0.044 ± 0.023
	CFAB-ATM	Time	279.4 ± 123.5	309.1 ± 188.6
		Rate	0.041 ± 0.017	0.034 ± 0.020
ADCS ADL-PI	Participant-report		1.17 ± 0.16	1.22 ± 0.23
	Study partner-report		1.09 ± 0.14	1.39 ± 0.31
SSPP-IADL	Participant-report		0.31 ± 0.37	0.77 ± 0.53
	Study partner-report		0.31 ± 0.50	0.89 ± 0.98

Values represent mean ± standard deviation

*ADCS ADL-PI* Alzheimer’s Disease Cooperative Study activities of daily living prevention instrument, *APT* Automated Phone Task, *ATM* automated teller machine, *CFAB* Czaja Functional Assessment Battery, *CN* clinically normal, *MCI* mild cognitive impairment, *PCP* primary care physician, *SSPP-IADL* Subjective Study Partner and Participant-reported instrumental activities of daily living

**Table 3:** Spearman correlations comparing Harvard APT (adjusted time) with CFAB, ADCS ADL-PI, and SSPP-IADL across all participants and within CN and MCI participants separately

			All participants				CN				MCI			
			APT-Script		APT-PCP		APT-Script		APT-PCP		APT-Script		APT-PCP	
			*r*_s_	*p*	*r*_s_	*p*	*r*_s_	*p*	*r*_s_	*p*	*r*_s_	*p*	*r*_s_	*p*
CFAB (CN: *n* = 29; MCI: *n* = 17)	CFAB-Script	Time	0.17	0.26	0.25	0.10	0.09	0.63	0.12	0.52	0.27	0.29	0.39	0.13
		Rate	**−0.42**	0.004*	**−0.75**	< 0.001*	−0.29	0.13	**−0.67**	< 0.001*	**−0.70**	0.002*	**−0.85**	< 0.001*
	CFAB-ATM	Time	**0.34**	0.02*	**0.42**	0.005*	**0.39**	**0.04**	**0.42**	**0.03**	0.26	0.33	**0.60**	0.01*
		Rate	−0.28	0.06	**−0.53**	< 0.001*	−0.33	0.09	**−0.42**	**0.03**	−0.21	0.44	**−0.68**	0.004*
ADCS ADL-PI	Participant-report (CN: *n* = 24; MCI: *n* = 14)		0.08	0.62	0.27	0.11	0.13	0.56	0.34	0.11	−0.05	0.86	0.001	1.00
	Study partner-report (CN: *n* = 19; MCI: *n* = 10)		0.26	0.18	**0.47**	0.009*	0.15	0.53	0.13	0.60	0.55	0.10	**0.72**	**0.02**
SSPP-IADL	Participant-report (CN: *n* = 52; MCI: *n* = 7)		0.20	0.13	**0.46**	< 0.001*	0.26	0.06	**0.38**	0.005*	0.16	0.73	0.66	0.16
	Study partner-report (CN: *n* = 36; MCI: *n* = 3)		−0.02	0.93	**0.48**	0.002*	0.03	0.85	**0.52**	0.001*	−0.50	0.67	−1.00	–

Significant *p* values ≤ 0.05 (uncorrected) are shown in bold along with the corresponding *r* values

*ADCS ADL-PI* Alzheimer’s Disease Cooperative Study activities of daily living prevention instrument, *APT* Automated Phone Task, *ATM* automated teller machine, *CFAB* Czaja Functional Assessment Battery, *CN* clinically normal, *MCI* mild cognitive impairment, *PCP* primary care physician, *SSPP-IADL* Subjective Study Partner and Participant-reported instrumental activities of daily living

*p values ≤ 0.05 after false discovery rate correction for multiple comparisons

### Harvard APT vs. CFAB

**Fig. 1:**
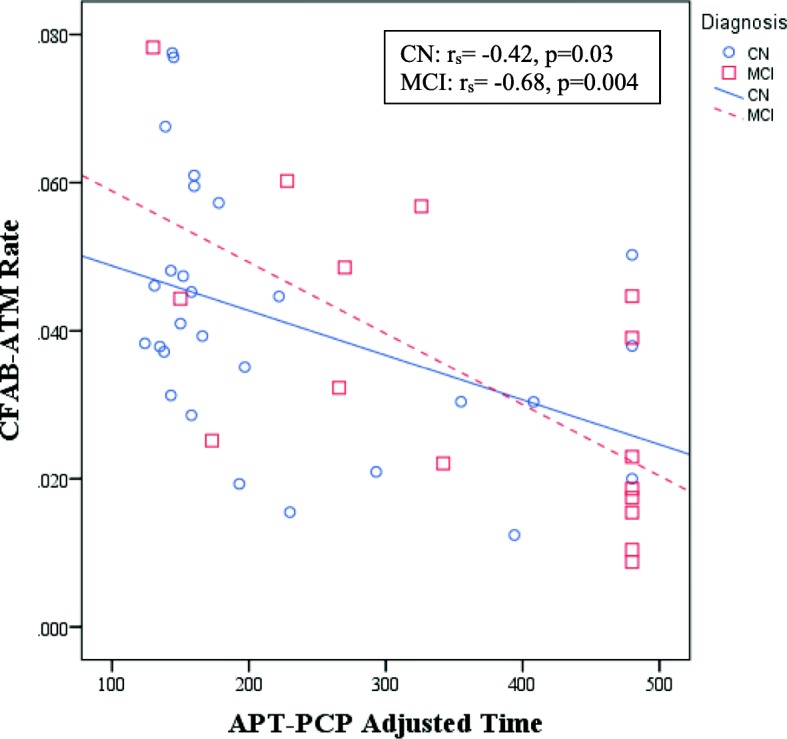
Scatter plot of unadjusted association between APT-PCP adjusted time and CFAB-ATM rate. Better performance on APT-PCP adjusted time was associated with better performance on CFAB-ATM rate in CN elderly and MCI participants. Lines represent ordinary least squares regression lines for each respective diagnostic group. *APT* Automated Phone Task, *ATM* automated teller machine, *CFAB* Czaja Functional Assessment Battery, *CN* clinically normal, *MCI* mild cognitive impairment, *PCP* primary care physician

Forty-six participants (29 CN and 17 MCI) performed both the Harvard APT and CFAB. With the general linear models, better performance on APT-Script adjusted time was associated with better performance on CFAB-Script (rate: unstandardized partial β = −685.2, 95% CI for β = −1057.3 to −313.1, *p* < 0.001) within each diagnostic group (but based on pooled data across diagnostic group). Better performance on APT-Script adjusted time was associated with better performance on CFAB-ATM (time: β = 0.09, 95% CI for β = 0.04 to 0.14, *p* = 0.002). Better performance on APT-PCP adjusted time was associated with better performance on CFAB-Script (rate: β = −4082.7, 95% CI for β = −5507.7 to −2657.8, *p* < 0.001). Better performance on APT-PCP adjusted time was associated with better performance on CFAB-ATM (time: β = 0.37, 95% CI for β = 0.13 to 0.60, *p* = 0.003; rate: β = −3615.6, 95% CI for β = −5474.8 to −1756.4, *p* < 0.001) (see Fig. 1). In preliminary tests, there was no significant interaction between CFAB-Script or CFAB-ATM and diagnosis in any of these models (APT-Script vs. CFAB-Script time: time: β = 0.04, *p* = 0.64; rate: β = 646.9, *p* = 0.08; APT-Script vs. CFAB-ATM time: time: β = −0.09, *p* = 0.10; rate: β = 131.3, *p* = 0.79; APT-PCP vs. CFAB-Script time: time: β = −0.46, *p* = 0.25; rate: β = 1382.5, *p* = 0.34; APT-Script vs. CFAB-PCP time: time: β = 0.07, *p* = 0.76; rate: β = 1595.0, *p* = 0.40). Therefore, it was removed and the model was rerun with main effects only.

### Harvard APT vs. ADCS ADL-PI

**Fig. 2:**
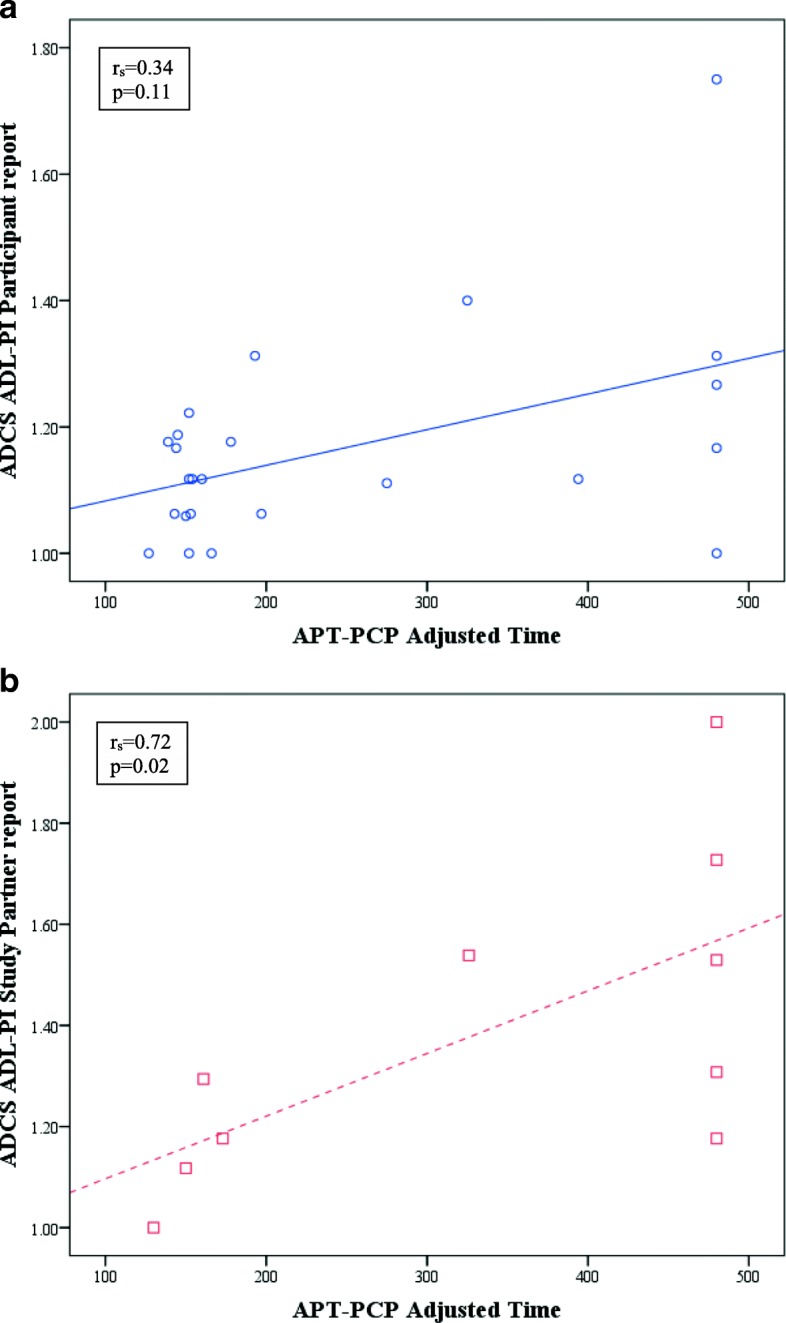
Scatter plot of unadjusted association between APT-PCP adjusted time and participant-reported and study partner-reported ADCS ADL-PI. **a** In CN participants, better participant-reported IADL were associated with better performance on APT-PCP. **b** In MCI participants, better study partner-reported IADL were associated with better performance on APT-PCP. Lines represent ordinary least squares regression lines. *ADCS ADL-PI* Alzheimer’s Disease Cooperative Study activities of daily living prevention instrument, *APT* Automated Phone Task, *PCP* primary care physician

Thirty-eight participants (24 CN and 14 MCI) who performed the Harvard APT also provided participant (self) report of the ADCS ADL-PI and 29 study partners (19 CN and 10 MCI) provided study partner report. With the general linear models, there was a significant association between APT-Script and the interaction between study partner-reported IADL and diagnosis, such that in MCI participants better performance on APT-Script was associated with better study partner-reported IADL (β = 102.1, 95% CI for β = 52.8 to 151.5, *p* < 0.001), whereas the relation was nonsignificant and virtually flat among CN participants. For APT-PCP, there was a significant interaction between participant-reported IADL and diagnosis, such that in CN participants better performance on APT-PCP was associated with better participant-reported IADL (β = 508.0, 95% CI for β = 22.5 to 993.5, *p* = 0.04) (see Fig. 2a.), whereas the relation was nonsignificantly negative among MCI participants. Better performance on APT-PCP was associated with better study partner-reported IADL (β = 291.0, 95% CI for β = 46.7 to 535.4, *p* = 0.02), but there was no significant interaction between study partner-reported IADL and diagnosis.

### Harvard APT vs. SSPP-IADL scale

Fifty-nine participants (52 CN and 7 MCI) who performed the Harvard APT also provided participant report of the SSPP-IADL scale, and 39 study partners (36 CN and 3 MCI) provided study partner report. Using Spearman correlations, in CN participants better performance on APT-PCP was associated with better participant-reported IADL (*r*_s_ = 0.38, *p* = 0.005) and study partner-reported IADL (*r*_s_ = 0.52, *p* = 0.001), and there were no significant associations with APT-Script. In MCI participants there were no significant associations; however, the sample size was very small (participant report *n* = 7; study partner report *n* = 3). Across all participants, better performance on APT-PCP was associated with better participant-reported IADL (*r*_s_ = 0.46, *p* < 0.001) and study partner-reported IADL (*r*_s_ = 0.48, *p* = 0.002), and there were no significant associations with APT-Script (see Table 3).

After FDR correction, significant associations were still noted in CN participants for APT-PCP vs. participant-reported IADL, and across all participants for APT-PCP vs. participant-reported IADL and APT-PCP vs. study partner-reported IADL (see Table 3).

### Factor analysis of Harvard APT, CFAB, and ADCS ADL-PI variables

**Fig. 3:**
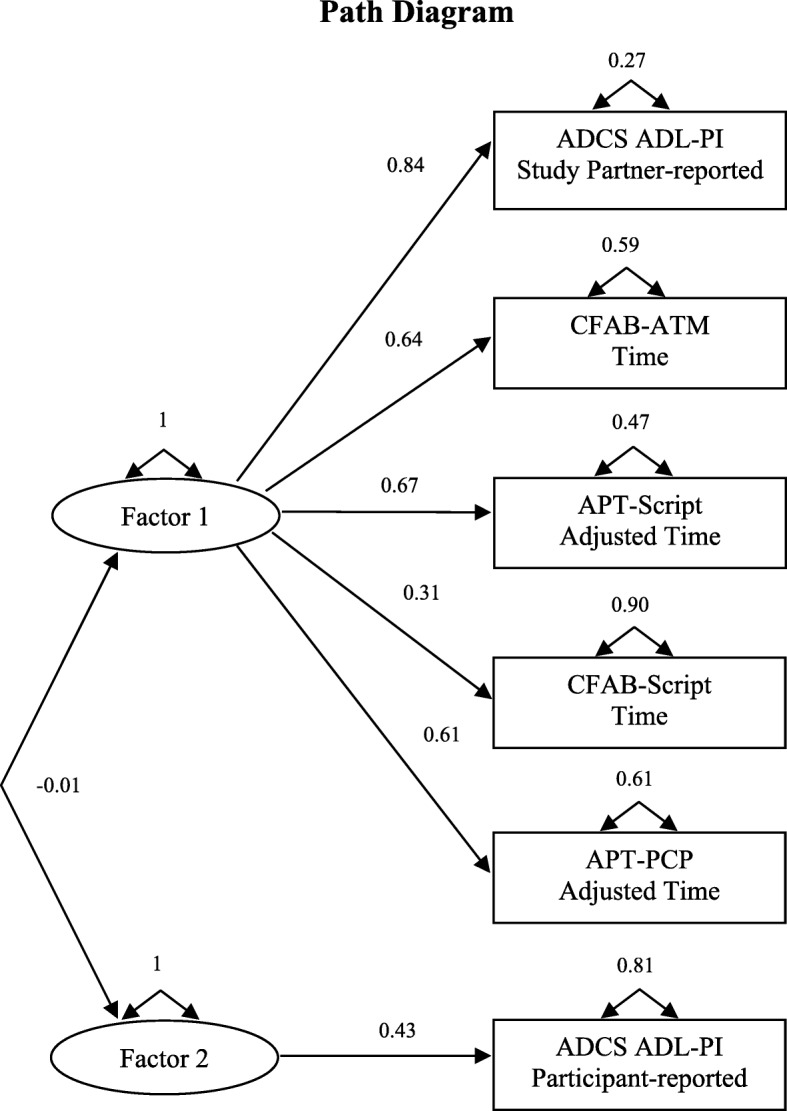
Factor analysis path diagram. The analysis included the following variables: APT-Script adjusted time, APT-PCP adjusted time, CFAB-Script time, CFAB-ATM time, participant-reported ADCS ADL-PI, and study partner-reported ADCS ADL-PI. The loadings of each variable onto the factors are displayed. The correlation between the factors is indicated by the double-headed curved arrow connecting the factors. *ADCS ADL-PI* Alzheimer’s Disease Cooperative Study activities of daily living prevention instrument, *APT* Automated Phone Task, *ATM* automated teller machine, *CFAB* Czaja Functional Assessment Battery, *PCP* primary care physician

The factor analysis provided evidence for one factor; that is, the Scree plot and variance measures were strongly suggestive of one predominant factor (variances = 2.04 for the first factor versus 0.3 for the second virtually uncorrelated factor), which was substantially loaded on (0.31 to 0.84) by all measures except the participant-reported ADCS ADL-PI (see Fig. 3). To further assess the dimensionality of these measures, we computed a Cronbach coefficient alpha, an internal consistency reliability measure that assesses unidimensionality, yielding a value of 0.67 when including all variables and 0.75 when excluding the participant-reported ADCS ADL-PI variable, indicating unidimensionality after removing that variable and consistent with the above factor analysis results.

## Discussion

In this small cross-sectional pilot study, we showed that the new ecologically valid Harvard APT, a novel phone performance-based IADL instrument designed to measure the earliest IADL changes in AD, appears to have good convergent validity. We demonstrated that in both CN and MCI participants better performance on the Harvard APT was associated with better performance on the CFAB, a computer-based measure of everyday functioning that has been validated in CN older adults, MCI, and older adults with schizophrenia. Additionally, better performance on the Harvard APT was associated with better IADL report on two subjective IADL assessments, specifically the established ADCS ADL-PI participant-reported portion in CN elderly and the study partner-reported portion in MCI, as well as the newly developed but yet to be validated SSPP-IADL scale participant-reported and study partner-reported portions in CN elderly. In showing that the Harvard APT related to other tests of IADL, we further support its construct validity as a test that is supposed to measure IADL. Finally, we also demonstrated a potential advantage for the Harvard APT over the subjective IADL scales in that direct assessment of the participant alone across CN elderly and MCI was adequate to measure IADL change using the Harvard APT, while with the established subjective scale, the ADCS ADL-PI, there was a differential response, albeit a modest effect in a small sample, between CN and MCI depending on who reported the symptoms (participant vs. study partner).

There is a growing body of literature suggesting that IADL changes can be captured at the stage of MCI or potentially even earlier [2, 3, 26, 27]. However, currently there are an inadequate number of sensitive measures to capture these early IADL changes. Recently, a roadmap for the necessary properties for an IADL measure that will capture changes at the stage of preclinical AD was described [28]. We believe that both the Harvard APT and CFAB, which are new performance-based IADL measures, demonstrate the ability to capture early changes in the amount of time it takes to complete a task, the accuracy with which the task is completed, and the complexity and granularity of the tasks performed as part of these IADL assessments. Both instruments are brief and do not require administration by an examiner (they are self-administered). However, the CFAB relies primarily on the computer (visual modality), while the Harvard APT relies primarily on the telephone (auditory modality), which is the most prevalent technology mode of communication in the elderly. Nearly all of the elderly in North America own a phone, while about a third own a computer which is used for the CFAB [9].

Multiple performance-based IADL tests have been developed and employed over the years. The psychometric properties of 21 such tests are summarized in a recent review article [11]. The majority of those tests target dementia, whereas few target MCI and even fewer target CN older adults at risk of AD. For MCI, the authors concluded that the Performance Assessment of Self-Care Skills and Direct Assessment of Functional Skills-Revised A, which assess multiple domains of IADL, and the Financial Capacity Instrument, which assesses a single IADL domain, are most suitable. These tests require administration or observation and scoring in person by a skilled examiner and take longer than 10 min to administer, and therefore may not be as easy to implement as the Harvard APT and CFAB, which can be self-completed and scored automatically. Moreover, the Harvard APT and CFAB are geared towards CN and MCI, as evidenced by the range of performance in these diagnostic groups.

While there are several advantages to using performance-based IADL tests as described above, there are also disadvantages; these tests, unlike subjective IADL scales, often capture a small number of activities that does not encompass all or most IADL, which is the case for the Harvard APT. Moreover, not all participants necessarily perform these activities in their daily life (i.e., regularly use an IVRS to refill a prescription). Finally, performance-based IADL tests may be confounded by visual or auditory impairment, the latter being especially pertinent for the Harvard APT. That said, the influence of auditory acuity is corrected for as part of the Harvard APT assessment, and participants without clinically significant visual or auditory impairment have been able to adequately complete the Harvard APT.

In the current study, we showed a significant association of moderate strength between the prescription refill tasks administered as part of the Harvard APT and CFAB. This suggests some equivalence in the modality of administration of these tasks (auditory vs. visual). Both tasks are relatively easy to perform and relate more closely to processing speed [7, 14]. We also demonstrated similar associations of moderate strength between the more complex tasks that relate to both processing speed and executive function (APT-PCP and CFAB-ATM) [7, 14].

Performance-based measures of everyday functioning are considered more objective and ecologically valid than traditional subjective IADL scales. However, subjective assessments can usually provide information on a wider range of activities and are more patient (participant) oriented since they rely on the report of the participant and/or study partner (informant) such as a family member or close friend. In the current study, we compared the Harvard APT to two such subjective IADL scales: 1) the ADCS ADL-PI, which has been validated, shown to be a sensitive assessment for early IADL changes, and is currently being used in secondary prevention trials in participants with preclinical AD [15]; and 2) the SSPP-IADL scale, which is a newly developed, yet to be validated instrument with items derived in a data-driven approach from existing sensitive IADL scales. We showed in the CN elderly that worse performance on the Harvard APT was associated with worse participant-reported IADL on the ADCS ADL-PI while, in MCI participants, worse performance on the Harvard APT was associated with worse study partner-reported IADL. This finding, although not robust and in a small sample, may be due to the decreasing awareness of cognitive and IADL deficits that develops as individuals become symptomatic and enter the stage of MCI and beyond, which has been described previously for cognitive function but not for IADL as far as we know [16, 29]. As such, using a sensitive performance-based instrument, such as the Harvard APT, may have an advantage when assessing IADL across a continuum of CN elderly and MCI or in individuals transitioning from CN to MCI because a single accurate assessment can be used without a concern about the source of information. Alternatively, it is possible that the differential correlation depending on diagnostic group between the Harvard APT and the ADCS ADL-PI participant vs. study partner report version may indicate that the Harvard APT is not as valid a test for this target population. Comparisons with larger samples and longitudinal follow-up will help determine that. When looking at the SSPP-IADL, worse performance on the Harvard APT was associated with worse participant and study partner-reported IADL. In these analyses, the overwhelming majority of participants were CN elderly. This suggests that the Harvard APT relates well to wider IADL performance as reported by the individual himself/herself in the absence of cognitive impairment and by a collateral who knows the individual well when the individual has MCI. However, since the SSPP-IADL has not been validated yet, its comparison to the Harvard APT is of limited value in establishing the convergent validity of the Harvard APT.

To further determine whether the IADL measures relate to each other (their dimensionality), we performed a factor analysis across all participants. We found that both Harvard APT tasks, both CFAB tasks, and study partner-reported ADCS ADL-PI score closely relate to each other, while participant-reported ADCS ADL-PI score did not relate to the other variables. The results of this analysis are in line with the rest of the analyses we performed, suggesting a simple association between the two performance-based measures (Harvard APT and CFAB) and a more complex association between the performance-based measure and subjective measure (Harvard APT and ADCS ADL-PI), which is influenced by the reporter of symptoms (participant vs. study partner). As mentioned above, the performance-based measures have the advantage of directly measuring performance as opposed to reporting an “opinion” on what an individual can or cannot do. It is notable that the ADCS ADL-PI participant report did not load on to the factor we found, suggesting that the participant report may represent a different construct than ability to perform IADL. It perhaps reflects other behaviors that may feed into ability to perform IADL, such as cognition, apathy, or depression.

The current study had several limitations. First, the number of participants completing both the Harvard APT and other IADL measures was relatively modest and consisted of two diagnostic groups. Moreover, there were two separate cohorts in the study—one cohort underwent the Harvard APT, CFAB, and ADCS ADL-PI, while the other cohort underwent the Harvard APT and SSPP-IADL. Therefore, these results will need to be replicated with larger samples, preferably across one cohort. Of note, previous analyses performed in larger samples has already shown that the Harvard APT (*n* = 207), CFAB (*n* = 147), and ADCS ADL-PI (*n* = 632) differentiate well between CN elderly and MCI participants [8, 14, 15]. Second, most of the correlations between the Harvard APT and other IADL tests in the study were modest to moderate in strength rather than moderate to strong as hypothesized. Again, replication in a larger sample is necessary to better determine the validity of these results. Third, this convenience sample consisted of participants with high levels of education and premorbid intelligence, not representative of the general population. However, our larger sample in prior reports on the Harvard APT did include a large number of minorities from underrepresented groups, especially among the CN elderly cohort (23%) [7, 8]. Finally, in this study we did not assess the relationship between the Harvard APT and AD biomarkers. In our initial validation study we were able to demonstrate an association between worse performance on the Harvard APT and inferior temporal atrophy in a small subset of participants [7]. Future larger studies using additional biomarkers such as amyloid and tau positron emission tomography imaging are needed. Such studies will help us assess the specific utility of the Harvard APT in participants with preclinical or prodromal AD.

## Conclusions

In this small pilot study, the Harvard APT appears to correlate well with other performance-based and subjective IADL measures targeting early-stage AD. These preliminary data provide good convergent validity for this new measure that augment prior validation efforts. The handful of sensitive IADL measures for early-stage AD have some but not all of the properties that make the Harvard APT especially compelling—it can be self-administered, it is brief, it provides time and error scores, it employs the modality most widely used by the elderly (telephone), it is ecologically valid, and it captures early IADL changes across the CN elderly and those with MCI. As secondary prevention trials in AD are being launched, there is a need for ecologically valid and reliable measures of early IADL changes. We hope to employ the Harvard APT or, when a computer-based visual assessment is preferable the CFAB, in future clinical trials of early-stage AD to determine whether these performance-based tools provide clinically meaningful outcomes for the interventions being tested.

## Additional file

Additional file 1: **Table S1.** FAQ, SIST-M, ECog, and new items comprising the SSPP-IADL. (DOCX 19 kb)

## Abbreviations

AD: Alzheimer’s disease
ADCS ADL-PI: Alzheimer’s Disease Cooperative Study activities of daily living prevention instrument
APT: Automated Phone Task
ATM: Automated teller machine
BWH: Brigham and Women’s Hospital
CDR: Clinical Dementia Rating
CFAB: Czaja Functional Assessment Battery
CI: Confidence interval
CN: Clinically normal
ECog: Everyday Cognition
FAQ: Functional Assessment Questionnaire
FCSRT: Free and Cued Selective Reminding Test
FDA: Food and Drug Administration
IADL: Instrumental activities of daily living
IVRS: Interactive voice response system
MADRC: Massachusetts Alzheimer’s Disease Research Center
MCI: Mild cognitive impairment
MGH: Massachusetts General Hospital
MMSE: Mini-Mental State Examination
PCP: Primary care physician
SIST-M: Structured Interview and Scoring Tool—Massachusetts Alzheimer’s Disease Research Center
SSPP-IADL: Subjective Study Partner and Participant-reported instrumental activities of daily living
