# Supplementary material for: Measuring instrumental activities of daily living in non-demented elderly: a comparison of the new performance-based Harvard Automated Phone Task with other functional assessments
Source: Alzheimers Res Ther. 2019 Jan 10;11:4. doi: 10.1186/s13195-018-0464-x (PMC6329044; doi:10.1186/s13195-018-0464-x)
Supplement: Supplementary file 1 — Table S1. FAQ, SIST-M, ECog, and new items comprising the SSPP-IADL. (DOCX 19 kb) [file 13195_2018_464_MOESM1_ESM.docx]

**Additional file: Marshall et al. Measuring instrumental activities of daily living in non-demented elderly: A comparison of the new Harvard Automated Phone Task to other functional assessments**

**Additional Table.** FAQ, SIST-M, ECog, and new items comprising the SSPP-IADL.

| **SSPP-IADL item** | **CN vs. MCI** | | **CN progression to MCI** | |
| --- | --- | --- | --- | --- |
|  | **p-value** | **Scale** | **p-value** | **Scale** |
| 1. Remembering appointments, family occasions, holidays, medications | <0.0001 | FAQ, ECog |  |  |
| 2. Remembering a few shopping items without a list | <0.0001 | ECog |  |  |
| 3. Paying attention to and understanding a TV program, book, or magazine |  |  | 0.0003 | FAQ |
| 4. Heating water, making a cup of coffee, turning off the stove |  |  | 0.0006 | FAQ |
| 5. Participating in games that involve retrieving words (e.g. Scrabble, crossword puzzles) | 0.0001 | SIST-M |  |  |
| 6. Navigating to unfamiliar areas | 0.001 | SIST-M |  |  |
| 7. Keeping mail and papers organized | 0.002 | ECog | 0.07 | ECog |
| 8. Developing a schedule in advance of anticipated events | 0.007 | ECog |  |  |
| 9. Fixing things or finishing projects (e.g. painting, repairing furniture, fixing appliances, plumbing work, yard work) | 0.002 | SIST-M |  |  |
| 10. Writing checks, paying bills, or balancing checkbook | 0.02 | ECog | 0.004 | FAQ |
| 11. Assembling tax records, business affairs, or other papers | <0.0001 | FAQ |  |  |
| 12. Performing mental tasks involved in former primary job | 0.002 | SIST-M |  |  |
| 13. Performing at work (if not retired) or volunteer position |  | | | |
| 14. Ability to hold positions of leadership in community or faith-based organizations |  |  |  |  |
| 15. Navigating an automated phone menu |  |  |  |  |
| 16. Using automated teller services (ATM) |  |  |  |  |
| 17. Using a cell phone |  |  |  |  |
| 18. Using a computer |  |  |  |  |

CN (clinically normal elderly), ECog (Everyday Cognition), FAQ (Functional Assessment Questionnaire), MCI (mild cognitive impairment), SIST-M (Structured Interview and Scoring Tool— Massachusetts Alzheimer’s Disease Research Center), SSPP-IADL (Subjective Study Partner and Participant-reported Instrumental Activities of Daily Living).
